# Response to the Fernández-Niño Comments on Hernández-Alvarado et al. Increase in Suicide Rates by Hanging in the Population of Tabasco, México between 2003 and 2012. *Int. J. Environ. Res. Public Health* 2016, *13*, 552

**DOI:** 10.3390/ijerph13070672

**Published:** 2016-07-01

**Authors:** Mervyn Manuel Hernández-Alvarado, Thelma Beatriz González-Castro, Carlos Alfonso Tovilla-Zárate, Ana Fresán, Isela E. Juárez-Rojop, María Lilia López-Narváez, Mario Villar-Soto, Alma Genis-Mendoza

**Affiliations:** 1 División Académica Multidisciplinaria de Comalcalco, Universidad Juárez Autónoma de Tabasco, Comalcalco, Tabasco 86650, Mexico; dramarilit@hotmail.com; 2 División Académica de Jalpa de Méndez, Universidad Juárez Autónoma de Tabasco, Cunduacán, Tabasco 86205, Mexico; thelma.glez.castro@gmail.com; 3 Subdirección de Investigaciones Clínicas, Instituto Nacional de Psiquiatría Ramón de la Fuente Muñiz, Mexico city 14370, México, fresan@imp.edu.mx; 4 División Académica de Ciencias de la Salud, Universidad Juárez Autónoma de Tabasco, Villahermosa, Tabasco 86150, Mexico; iselajuarezrojop@hotmail.com; 5 Hospital General de Yajalón, Secretaría de Salud, Yajalón, Chiapas 29930, Mexico; lilialopez03@hotmail.com; 6 Hospital de Alta Especialidad “Gustavo A. Rovirosa P”, Villahermosa, Tabasco 86020, Mexico; mariovillarsoto@hotmail.com; 7 Instituto Nacional de Medicina Genómica (INMEGEN), Servicios de Atención Psiquiátrica (SAP), Secretaría de Salud, Mexico city 14610, Mexico; adgenis@inmegen.gob.mx

We thank the comments of Fernández-Niño [1] addressing our article [2] “Increase in Suicide Rates by Hanging in the Population of Tabasco, Mexico between 2003 and 2012”, which pointed out that the use of the epidemiological concept “prevalence” is not correctly applied in the present manuscript.

In the discussion section we wrote: “However, this difference could be explained considering the fact that Tabasco State in Mexico has a historic higher prevalence of suicide than the rest of the country. However, the suicide prevalence remained unchanged in females.” This should have said: “However, this difference could be explained considering the fact that Tabasco State in Mexico historically has a higher incidence of suicide than the rest of the country. However, the suicide incidence remained unchanged in females”.

In the paper mentioned above we showed changes in the incidence of suicide in the population of Tabasco, Mexico, between 2003–2012. We identified a specific age group where the incidence of suicide increased 180% from 2003–2012; however, the use of the word “prevalence” was inappropriate as Fernández-Niño clearly noticed and explained [1].

We appreciate this valuable comment; we agree it is very important to have adequate use of the epidemiological terminology, and we will make sure that in future reports the correct terminology is used.
